# Continuous salt stress-induced long non-coding RNAs and DNA methylation patterns in soybean roots

**DOI:** 10.1186/s12864-019-6101-7

**Published:** 2019-10-12

**Authors:** Rui Chen, Ming Li, Huiyuan Zhang, Lijin Duan, Xianjun Sun, Qiyan Jiang, Hui Zhang, Zheng Hu

**Affiliations:** 1 Tianjin Institute of Agricultural Quality Standard and Testing Technology, Tianjin Academy of Agricultural Sciences, Tianjin, 300381 China; 2 The National Key Facilities for Crop Genetic Resources and Improvement, Institute of Crop Sciences, Chinese Academy of Agricultural Sciences, Beijing, 100081 China; 3 Zhengzhou Fruit Research Institute, Chinese Academy of Agricultural Sciences, Zhengzhou, 450009 China

**Keywords:** Whole transcriptome sequencing, DNA methylation, Continuous salt stress, Long non-coding RNAs, Soybean roots

## Abstract

**Background:**

Environmental stimuli can activate a series of physiological and biochemical responses in plants accompanied by extensive transcriptional reprogramming. Long non-coding RNAs (lncRNAs), as versatile regulators, control gene expression in multiple ways and participate in the adaptation to biotic and abiotic stresses.

**Results:**

In this study, soybean seedlings were continuously cultured for 15 days with high salinity solutions started from seed germination. Strand-specific whole transcriptome sequencing and stringent bioinformatic analysis led to the identification of 3030 long intergenic non-coding RNAs (lincRNAs) and 275 natural antisense transcripts (lncNATs) in soybean roots. In contrast to mRNAs, newly identified lncRNAs exhibited less exons, similar AU content to UTRs, even distribution across the genome and low evolutionary conservation. Remarkably, more than 75% of discovered lncRNAs that were activated or up-regulated by continuous salt stress mainly targeted proteins with binding and catalytic activities. Furthermore, two DNA methylation maps with single-base resolution were generated by using reduced representation bisulfite sequencing, offering a genome-wide perspective and important clues for epigenetic regulation of stress-associated lncRNAs and protein-coding genes.

**Conclusions:**

Taken together, our findings systematically demonstrated the characteristics of continuous salt stress-induced lncRNAs and extended the knowledge of corresponding methylation profiling, providing valuable evidence for a better understanding of how plants cope with long-term salt stress circumstances.

## Background

RNA molecules play vital roles in genetic information delivery and gene expression regulation during various life processes. Long non-coding RNAs (lncRNAs) are generally longer than 200 nucleotides (nt), containing capped 5′-ends, spliced introns and poly(A) tails, but lack protein-coding capability [1]. LncRNAs are mainly located in the cytoplasm and transcribed by different RNA polymerases (II, III, IV and V) [2]. Compared with messenger RNAs (mRNAs), lncRNAs are expressed at very low levels but widespread across the genome [3, 4]. Based on their origins and biogenesis, lncRNAs could be classified into three categories: (a) long intergenic ncRNAs (lincRNAs) derived from intergenic regions, (b) intronic ncRNAs (incRNAs) produced from introns and (c) natural antisense transcripts (lncNATs) transcribed from the opposite strands of protein-coding genes [5].

Animal lncRNAs have been extensively studied and proven to be functional in essential biological processes, such as cell cycle control [6], immune surveillance [7], stem cell differentiation [8], development and diseases [9]. In plants, taking advantage of next-generation sequencing (NGS) technologies, large batches of lncRNAs have been identified in the recent 5 years [4, 10–22]. In soybean, 6018 lincRNAs have been recently identified by using previously reported transcriptomic data [23]. Although the samples came from different tissues and developmental stages, these lincRNAs only represented the group of poly(A)-containing lincRNAs under normal circumstances. Growing evidence indicated that plant lncRNAs are involved in the processes of vernalization [24], male sterility [25], photomorphogenesis [26], phosphate homeostasis [27] and alternative splicing [28]. So far, functional roles of plant lncRNAs remain largely unknown, even though tens of thousands of lncRNAs had been discovered.

Cytosine DNA methylation is a heritable epigenetic mechanism and widespread in eukaryotes. The pattern of DNA methylation is biased and dynamic which controls gene expression and regulates environmental adaptation and genome evolution [29]. According to the cytosine contexts, DNA methylation is classified into three categories: CG, CHG, and CHH (H is either A, T, or C). Several DNA methyltransferases are responsible for catalyzing and maintaining the state of DNA methylation, including Methyltransferase 1 (MET1), Chromomethylase 3 (CMT3), Domain Rearranged Methyltransferase 2 (DRM2) and CMT2 [30]. It is well known that both lncRNAs and small RNAs could guide DNA methylation or histone modifications and silence target genes, which was defined as RNA-directed DNA methylation (RdDM) pathway [31]. Genome-wide investigation of the DNA methylation status would contribute to a better understanding of the regulatory roles of lncRNAs.

In this study, we focused on exploring continuous salt stress-induced lncRNAs as well as their potential functional roles in soybean roots. Whole transcriptome and methylation sequencing strategies were combined and performed, resulting in a more comprehensive view of regulatory networks underlying plant adaptations to long-term salt stress. Our results shed light on the complexity and diversity of plant lncRNAs, which would benefit the molecular improvement of soybean in the future.

## Results

### Genome-wide identification of lncRNAs

**Fig. 1:**
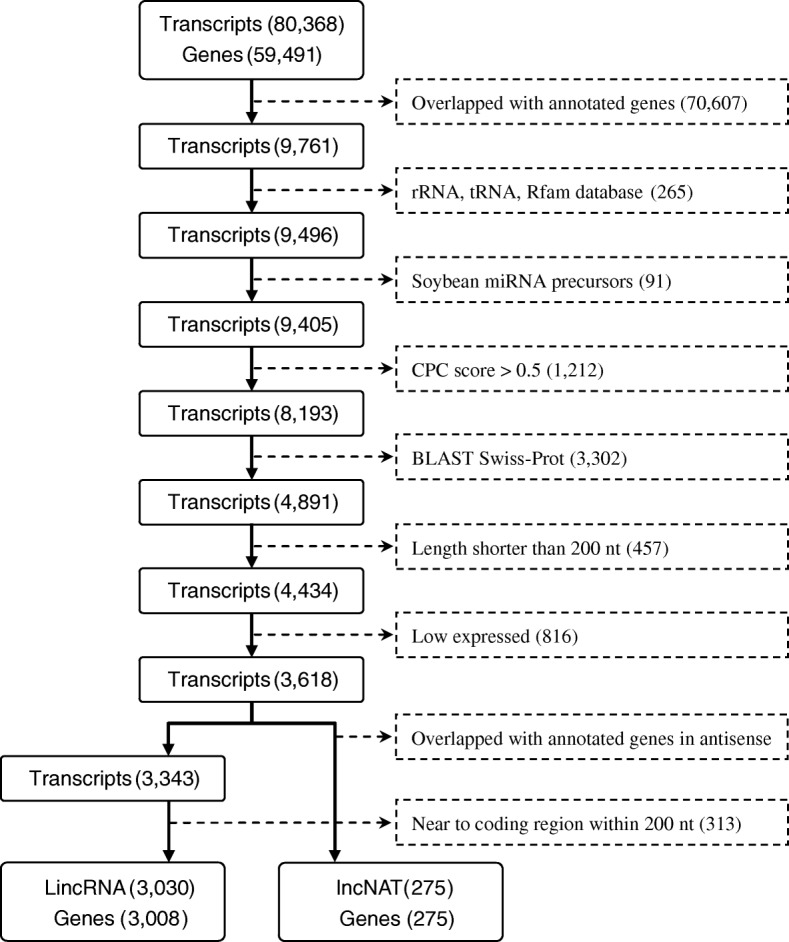
Flowchart of lncRNA identification in soybean roots. Transcripts were subject to a series of steps for excluding known protein-coding RNAs, house-keeping RNAs, and miRNA precursors. Besides, CPC program and Swiss-Prot database were used for accessing their coding potentials and further filtration. Low expressed (FPKM < 2) and short transcripts (< 200 nt) were also discarded. At last, transcripts located on the opposite strands of known genes were classified as lncNATs, while novel intergenic transcripts 200 nt away from coding regions were regarded as lincRNAs

To systematically identify continuous salt responsive lncRNAs in soybean roots, whole transcriptome sequencing based on rRNA-depletion strategy was performed and generated 44,672,549 and 42,417,198 strand-specific paired-end reads under control (WT) and continuous high salinity (SA) conditions, respectively (Additional file 7: Table S1). After removing low quality reads (~ 1.3%) for each library, clean reads were combined and aligned onto the soybean genome, resulting in the discovery of 80,368 transcripts corresponding to 59,491 genes in total. Based on the results of genomic mapping, a stringent bioinformatic pipeline was developed for accurately recognizing lincRNAs and lncNATs. According to the annotation information, 70,607 transcripts were found to be overlapped with known genes in the same strand which were designated as mRNAs and excluded for further analysis. Subsequently, several steps were carried out for the removal of various house-keeping RNAs (265) and microRNA (miRNA) precursors (91). To guarantee the complete elimination of protein-coding genes, two approaches were applied in order. First, Coding Potential Calculator (CPC) was used to evaluate the coding potential for each transcript and those with a score > 0.5 were discarded (1212). Second, the remaining transcripts were aligned against the Swiss-Prot database and any hitted transcript was also excluded (3302). Furthermore, transcripts shorter than 200 nt (457), low-expressed with FPKM < 2 (816) or immediately adjacent to protein-coding genes in the same strand (313), were removed in turn. Finally, 3305 high confidence lncRNA candidates were identified including 3030 lincRNAs and 275 lncNATs, corresponding to 3008 and 275 genes, respectively (Fig. 1; Additional files 9 and 10: Table S3 and S4).

To validate the authenticity of discovered lncRNAs, 22 lincRNAs were randomly chosen from 1300 repetitive region-related lincRNAs for reverse transcription PCR (RT-PCR) experiment, which represented potential fake candidates due to their origin and low abundance. As a result, all 22 lincRNAs could be correctly amplified except for lincRNA_1231 whose length of PCR product was larger than expected (Additional file 5: Figure S5). That might be caused by wrong genome assembly, RNA editing or primer specificity. PCR products of 12 lincRNAs were confirmed by Sanger sequencing. This RT-PCR result suggested that whole transcriptome sequencing approach and our bioinformatic pipeline for lincRNA identification were reliable and reproduced.

### Properties of discovered lncRNAs

**Fig. 2:**
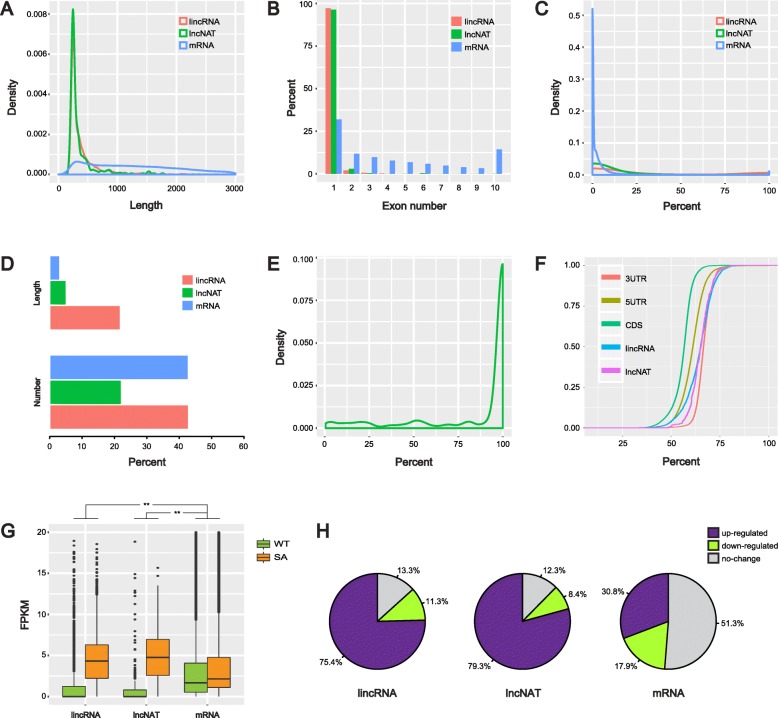
Characteristics of newly identified lncRNAs compared to mRNAs. Length (**a**) and exon number (**b**) distributions. Overlapped percentages with repetitive sequence in the soybean genome (**c** and **d**). Coverage rates between lncNATs and corresponding genes located on the opposite strands (**e**). Empirical cumulative distributions of AU content (**f**). Normalized expression values (**g**) and percentages of differentially expressed lncRNAs and mRNAs (**h**)

The characteristics of newly identified lncRNAs were comprehensively surveyed in contrast with mRNAs. In view of length, lncRNAs were significantly shorter than mRNAs. The majority of lncRNAs ranged from 200 to 500 nt, and the average length of lincRNAs and lncNATs were 375 nt and 358 nt, respectively, while the average length of mRNAs was 1303 nt, almost four times larger than those of lncRNAs (Fig. 2a). Compared with the previous report in soybean, lincRNAs and mRNAs in our study were 0.8 times and 0.29 times shorter in length, which might be attributed to tissue specificity and long-term high-salinity environment [32]. For lncNATs, approximately 72.4% in number was entirely transcribed from the opposite strands of corresponding genes, which could form double-stranded RNA duplexes and produce functional siRNAs for a broad range of downstream regulations (Fig. 2e). Exon number analysis showed that 97.2% of lincRNAs and 96.4% of lncNATs were composed of one single exon, whereas it was only 31.9% for mRNAs (Fig. 2b). This pattern was consistent with previous studies in soybean [32], chickpea (*Cicer arietinum*) [33], maize (*Zea mays*) [10] and cucumber (*Cucumis sativus*) [19].

Investigation of whether a transcript is derived from repetitive sequences would contribute to better understanding of its biogenesis and function. For lincRNAs, 42.9% in number was overlapped with or completely originated from repetitive regions, corresponding to 21.8% in total length. A similar percentage was observed for mRNAs in number (42.8%), however only 3.13% was counted in total length. LncNATs were poorly correlated to repetitive regions which were 22.2% in number and 5.1% in total length (Fig. 2c and d). A number of novel lincRNAs generated from repeat sequences under continuous salt stress indicated that the repetitive region plays a crucial regulatory role during stress responses and plant adaptations.

The empirical cumulative distribution of AU content was analyzed for all transcripts, where mRNAs were split into 5′-UTR, CDS and 3′-UTR partitions and separately calculated (Fig. 2f). As a result, 3′-UTR had the highest AU content, while CDS was the lowest and 5′-UTR occupied the middle position. Interestingly, lincRNAs and lncNATs demonstrated an intermediate phase between 3′-UTR and 5′-UTR, suggesting their constitutional and potential functional similarities with UTRs. Expression profiling analysis revealed that most of lincRNAs and lncNATs were remarkably induced by continuous salt stress in contrast to mRNAs (Chi-square test, *P* < 0.001). The medians of FPKM for lincRNAs, lncNATs and mRNAs under SA/WT conditions were 4.39/0, 4.99/0 and 2.36/1.92, respectively (Fig. 2g). In terms of number, 75.4% of lincRNAs and 79.3% of lncNATs were activated or up-regulated more than 2-fold, while there was only 30.8% for mRNAs (Fig. 2h and Fig. 4c). Unlike mRNAs, it is obvious that lincRNAs and lncNATs are very closely related to continuous salt stress and probably participate in complex regulatory networks for rescuing plant from such an extreme environment.

### Genome distribution of identified lncRNAs

**Fig. 3:**
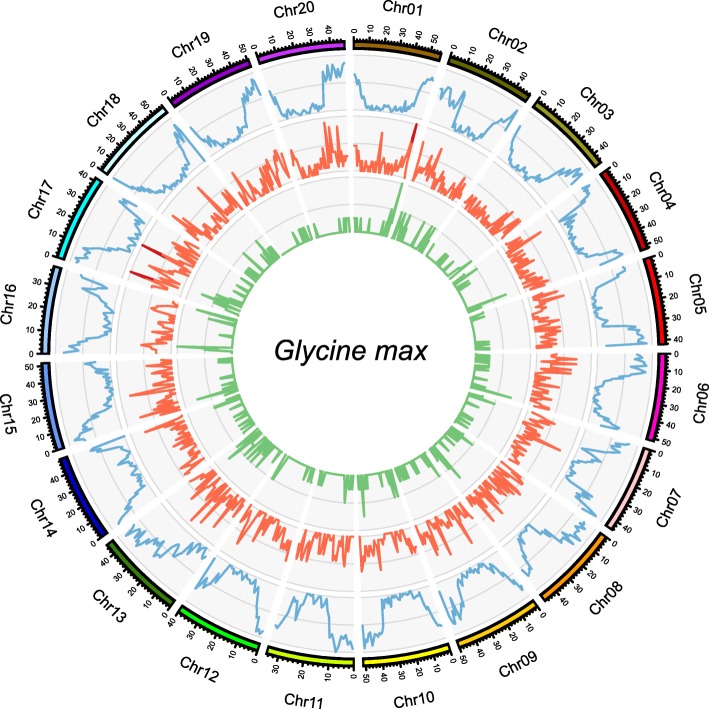
Chromosomal distributions of transcripts across the soybean genome. Blue, red and green lines indicate mRNA, lincRNA and lncNAT, respectively. Window size is 1 Mb. For each section, vertical coordinates indicate the numbers of transcripts

Landscapes of lincRNAs showed no obvious bias across the soybean genome. The maximum was 184 on Chr17 and the minimum was 108 on Chr11. For each chromosome, lincRNAs were evenly distributed including the centromeric region. In contrast, mRNAs displayed a preferential distribution in centromere-distant areas. LncNATs were basically in accordance with mRNAs because of their inherent linkage (Fig. 3). The distinct patterns of genomic distribution implied their functional differentiation, also supporting the notion that lincRNAs could act as global regulators and involve in centromere maintenance [34].

To globally compare the expression profiling under different conditions, genomic mapping and transcriptome assembly were rerun using two separate datasets. As a result, total numbers of expressed loci were found to be 41,951 (WT) and 63,753 (SA), and the average length of transcripts was 1444 nt (WT) and 1186 nt (SA), respectively (Additional file 8: Table S2). Gene number increasing more than 50% and transcript length declining simultaneously indicated that the transcriptome in soybean roots was dramatically reconstructed by continuous salt stress not only for lncRNAs but also for protein-coding genes.

### Sequence homology to transposable elements (TEs)

**Fig. 4:**
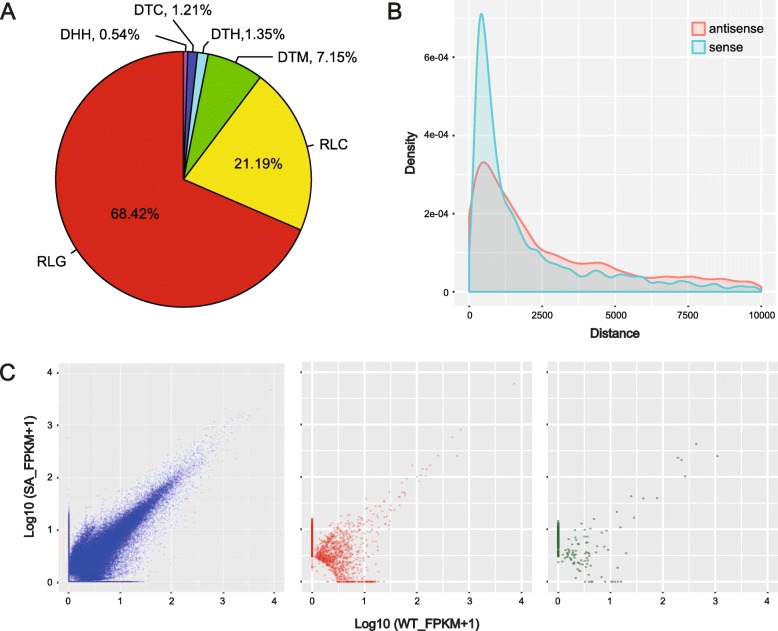
Features of newly identified lncRNAs. Compositions of TEs in lincRNAs (**a**). RLG, LTR *Gypsy*; RLC, LTR *Copia*; DTM, Mutator; DTH, PIF-Harbinger; DTC, CACTA; DHH, Helitron. Distance distribution between lincRNAs and their nearest genes (**b**). Expression values for mRNAs (blue), lincRNAs (red), and lncNATs (green) under different conditions (**c**). Vertical and horizontal coordinates indicate values under continuous salt stress and control conditions, respectively

In soybean genome, TEs are enriched in the centromeric regions and contribute to gene evolution and genome shaping [35]. Comparative analysis against the soybean TE database [36] showed that 24.5% of lincRNAs in number harbored TEs. Of these, 87.3% of length was occupied by TEs in average. However, TE-overlapped mRNAs and lncNATs were only 6.8 and 3.3% in number. Further analysis of lincRNA-overlapped TEs showed that, 89.6% was derived from Class I retrotransposon (LTR *Gypsy*: 68.4%; LTR *Copia*: 21.2%), and DNA transposon only accounted for 10.4%, which was similar with the original proportions of TEs in soybean genome [37] (Fig. 4a; Additional file 11: Table S5). Long terminal repeat (LTR) retrotransposons had been reported to be responsible for the production of lncRNAs [38], implying that feedback regulations might exist in the biogenesis of lincRNAs like miRNAs.

### Functional analysis of lincRNAs and lncNATs

Previous studies elucidated that regulating neighbor protein-coding genes in *cis* was the major mode for lincRNA function [39]. To address this, a genome-wide scan was performed to find neighbor genes proximal to lincRNAs in either sense or antisense orientation. As a result, 3002 nearest genes were found and regarded as putative targets of lincRNAs (Additional file 9: Table S3). Of these, 62.1% were located in the same strand and 37.9% came from the antisense strand. The distance between lincRNAs and their target genes ranged from 200 bp to 400 kb. Nearly half of them were close to lincRNAs within 2.5 kb (Fig. 4b). For lncNATs, corresponding genes located in the opposite strands were collected as their targets. All target genes of lincRNAs and lncNATs were subject to Gene Ontology (GO) analysis to determine their functions. Although no signicant GO term was identified for enrichment analysis, GO analysis showed highly similar results between lincRNAs and lncNATs, in which catalytic activity (GO:0003824) and binding (GO:0005488) were major GO terms in the Molecular Function (MF) category (Additional file 2: Figure S2). Similar functions of target genes for lincRNAs and lncNATs implied their inherent connections, even though these regulations happened in different ways.

### Conservation analysis of identified lncRNAs

To achieve a more comprehensive result, 66 plant genomes were downloaded and used as backgrounds to analysis the sequence conservation of newly identified lncRNAs. As expected, most of lincRNAs (99%) and all of the lncNATs (100%) had homologs in wild soybean (*Glycine soja*). However, 92.5% of lincRNAs and 86.5% of lncNATs disappeared in common bean (*Phaseolus vulgaris*) genome. Only ~ 3% of lincRNAs and lncNATs possess homologs in chickpea (*C. arietinum*), clover (*Trifolium pratense*) and caliph medic (*Medicago truncatula*) genomes and they are very scarce in other distantly related plant genomes (Additional file 3: Figure S3). Comparing to protein-coding genes, lncRNAs are highly species-specific and have a very low level of inter-specific conservation which had been demonstrated in soybean and other plants [40]. Meanwhile, the absence of conserved lncRNA candidates among plant species reflected the fact that the physiological origins and molecular functions of lncRNAs might be distinct from other regulatory non-coding RNAs, such as miRNAs.

### DNA methylation levels of chromosomes and individual genes

Bisulfite sequencing was carried out and generated approximately 26-fold and 21-fold raw reads from soybean roots exposed to control and continuous salt stress conditions (Additional file 7: Table S1). Genomic mapping efficiencies of Bismark program were ~ 66%. In-depth analysis of 2,161,369,408 (WT) and 1,725,745,072 (SA) cytosine sites for three types of contexts: CpG, CHG, and CHH, yielded two strand-specific DNA methylation profiling with single-base resolution. Overall, 63.7% of CpGs, 43.6% of CHGs and 4% of CHHs were methylated under control, which was slightly higher than those under continuous salt stress (Additional file 12: Table S6).

**Fig. 5:**
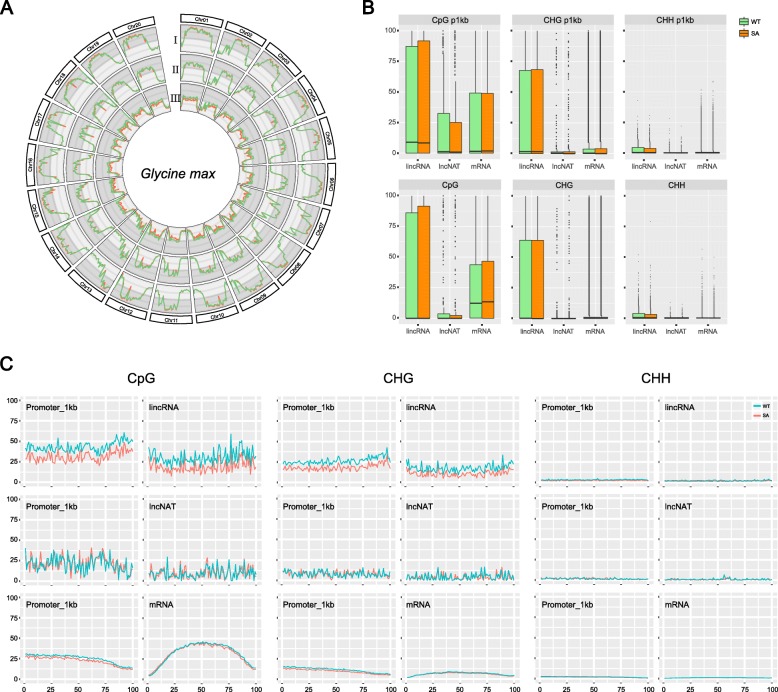
Cytosine methylation statuses for chromosomes and transcripts. Distributions of RMCs for CpGs (I), CHGs (II), and CHHs (III) across the soybean genome (**a**). Vertical coordinates range from 0 ~ 100% for CpGs and CHGs, and 0 ~ 10% for CHHs. Box plots of RMCs for transcripts and their promoter regions (upstream 1 kb) (**b**). Length-wise distribution of RMCs along transcripts and their promoter regions (upstream 1 kb) (**c**)

The ratios of methylated cytosines (RMCs) were calculated and used as the key indicator to compare different methylation states. For all types of contexts, chromosomal distributions of RMCs displayed peaks near centromeres and valleys at telomeres. In the middle of each chromosome, RMCs of CpGs maintained a very high level (~ 85%), while those of CHGs and CHHs were around 65 and 5%, respectively. Overall, salt-induced alterations of RMCs were very slight across chromosomes (Fig. 5a).

The RMCs of individual genes and their promoter regions (upstream 1 kb) were also investigated and plotted including lncRNAs and mRNAs (Additional files 13, 14 and 15: Table S7 - S9). Above all, 44.3% of lincRNAs, 63.4% of lncNATs and 17.5% of mRNAs were never methylated for CpGs and CHGs. No clear difference could be found between different conditions for methylation levels of lncRNAs and mRNAs on the whole. However, a distinguishable difference could be seen between promoter and gene regions for CpGs, where the methylation levels were higher in promoters for lincRNAs but mRNAs presented the opposite situation (Fig. 5b). Similar to the expression level, CpG methylation ratios of lncRNAs varied more dramatically by continuous salt stress in contrast to their target mRNAs (Additional file 6: Figure S6 and Additional file 16: Table S10). It could be speculated that lncRNAs tended to be more sensitive at both transcriptomic and epigenetic levels in response to environmental change, which were preferentially altered and led to a broad range of downstream regulatory events.

In order to compare the positional difference of methylation levels within gene, the length-wise distribution of RMCs was separately calculated and diagrammed. For CpGs and CHGs, RMCs of lincRNAs were obviously decreased due to continuous salt stress. Also, slight drops could be seen in the promoter regions of mRNAs. If carefully looking at the RMCs of mRNAs in CpGs, an N-shape curve and a slight forward shift could be found, suggesting that continuous salt stress aggravated the methylation status of mRNAs in the front part and alleviated it at back (Fig. 5c).

To visualize the differentially methylated genes under different conditions, the ratios of RMCs were computed for each gene and shown as scatter diagrams (Additional file 4: Figure S4A). No apparent difference could be found between promoter and coding regions. Base composition analysis for CHG and CHH contexts showed that A and T were preferential bases in the “H” position (Additional file 4: Figure S4B). All these results illustrated the effect of continuous salt stress on DNA methylation profiling, which was instructive to the future study of stress-associated epigenetic regulations.

## Discussion

Soybean (*Glycine max*) is one of the most important crops for edible oil and protein production worldwide. Salinity stress is the major limiting factor for soybean growth and productivity [41]. To simulate the actual environment, soybean seedlings were continuously cultured with the same concentration of NaCl solutions which was started from seed germination. With the aid of ‘omics’ technologies, the repertoire of transcripts was comprehensively investigated in soybean roots under different conditions. As a result, the total number of expressed genomic loci was increased by more than 50% in response to continuous salt stress. Meanwhile, ~ 77% of identified lncRNAs were activated or up-regulated more than 2-fold, illustrating that long-term high-salinity environment fundamentally reshaped the soybean transcriptome, especially for lncRNAs.

In order to accurately recognize lncRNAs, a stringent bioinformatic pipeline was set up and resulted in the identification of 3030 lincRNAs and 275 lncNATs in soybean roots. Very recently, Golicz et al. discovered 6018 lincRNA genes in soybean via re-analysing poly(A) RNA-seq data collected from various tissues at different developmental stages [32]. Compared with these reported lincRNAs, 87.7% of newly identified lincRNAs in our study came from novel loci. In other words, a large number of lncRNAs might not express under normal condition, which could be specially activated by constant high-salinity stress. Considering the temporal and spatial patterns of gene expression, it is reasonable to believe that the repertoire of authentic lncRNAs was seriously underestimated. Functional analysis showed that proteins with binding and catalytic activities were major targets for these newly identified lncRNAs. Disparate biogenesis but similar targets for lincRNAs and lncNATs implied that they might work in a synergetic and complementary pattern.

UTR regions harbor various binding sites for regulators, controlling the stability, transportation and translational efficiency of transcripts [42]. In Arabidopsis, both 3′- and 5′-UTR regions of stress-responsive genes were seriously shortened at the whole-genome level [43]. In our results, the transcript shrinkage at different degrees induced by continuous salt stress was observed not only for mRNAs but also for lncRNAs. In terms of AU content, lncRNAs exhibited similarities to UTR regions, providing a reasonable explanation for their length reduction. Although the molecular mechanism behind this phenomenon is still unknown, it could be speculated that shrinking a wide repertoire of RNA molecules, as an intrinsic mechanism, might save metabolic energy and help plants to cope with stressful situation.

DNA methylation, as a conserved epigenetic mechanism, is closely associated with plant responses and adaptations to biotic and abiotic stresses. Until now, genome-wide investigation of salt-induced methylation profiling had been reported in rice [44], wheat [45], and caliph medic (*M. truncatula*) [46], but not yet for soybean. Utilizing more than 47-fold raw data from bisulfite sequencing, two DNA methylation maps with single-base resolution were generated from soybean roots under control and continuous salt conditions, providing a foundation for further analysis of methylation levels of individual genes. Recently, Yaish et al. studied the methylation status of root tissues in legume *M. truncatula* and found that ~ 75% of methylated sites were CHHs and the average methylation levels were increased for all cytosine contexts in response to salinity [46]. In our results, only ~ 19.5% of all methylated sites were CHHs, however methylated CpGs and CHGs occupied the majority as ~ 44% and ~ 36.5%, respectively (Additional file 12: Table S6). The paradox of changing tendency could be attributed to various factors, such as sampling, treatment, species difference, tissue and developmental specificities. In their study, *M. truncatula* samples were irrigated with 204 mM NaCl solution for 1 week at the pre-flowering stage (9-week-old), whereas our soybean samples were continuously cultured with 80 mM NaCl solution for 2 weeks from seed germination to seedlings. Despite the fact that cytosine DNA methylation is heritable mark among eukaryotes, more evidence supports that DNA methylation is highly dynamic and more complicated than previously realized [47]. Salt-induced alterations of DNA methylation in mRNAs, lincRNAs, lncNATs and their promoter regions provided valuable clues to figure out the effects of continuous salt stress on epigenetic regulations.

## Conclusions

Whole transcriptome sequencing and bioinformatic analysis led to the identification of 3030 lincRNAs and 275 lncNATs in soybean roots under control and continuous salt stress conditions. In contrast to mRNAs, identified lncRNAs were less exon-containing, mostly salt-inducible, similar AU composition to UTRs, evenly distributed across the genome, low evolutionarily conservative, and prone to regulate similar targets. Moreover, evidence from bisulfite sequencing sketched the contours of methylation profiling for soybean adaptation to long-term salt stress. Our results shed light on the sophisticated mechanisms underlying salt tolerance and provided important information for a better understanding of the regulatory roles of lncRNAs in soybean.

## Methods

### Plant material, salt treatment and DNA/RNA extraction

The seeds of cultivar Williams 82 used in this study were stored in the Stress-Tolerance Research Laboratory, Institute of Crop Sciences at Chinese Academy of Agricultural Sciences. Soybean seeds were germinated on MS medium in culture dishes with distilled water and 80 mmol/L NaCl. Approximately one hundred seedlings for each treatment were transplanted in Hoagland nutrient solution under continuous conditions. The whole cultivation process was accomplished in a growth chamber with a 14 h/10 h light/dark photoperiod at 20 °C. Root tissues of 15-day-old seedlings were collected and quickly frozen in liquid nitrogen until DNA/RNA isolation (Additional file 1: Figure S1). An optimized CTAB method was used for genomic DNA extraction. Total RNA was isolated using TRIzol reagent (Invitrogen) according to the manufacturer’s instructions and subsequently treated with RNase-free DNase I (Fermentas). The quantity and integrity of genomic DNA and total RNA were evaluated by Nanodrop spectrophotometer ND-1000 (Thermo Scientific) and 1% (w/v) agarose gel electrophoresis.

### Whole transcriptome sequencing

To realize the capture of both poly(A) and non-poly(A) RNAs, an rRNA-depleted approach combined with strand-specific RNA-Seq was adopted for transcriptome sequencing. TruSeq Stranded Total RNA Library Preparation Kit with Ribo-Zero™ Plant (RS-122-2401, Illumina) was used for library construction. The library concentration and quality were measured by Qubit 2.0 Fluorometer (Life Technologies) and Agilent 2100 Bioanalyzer (Agilent Technologies), respectively. Paired-end 126 bp strategy was used for Illumina sequencing.

### Reduced representation bisulfite sequencing

Methylation libraries were prepared using the NEXTflex Bisulfite-Seq Kit (5119-02, Bioo) including steps for end repair, adapter ligation, bisulfite conversion, and limited amplification. After purification and validation, libraries were sequenced with paired-end 126 bp strategy.

### LincRNA and lncNAT analysis

Raw reads from the whole transcriptome sequencing were firstly cleaned by Sickle with default parameters (https://github.com/najoshi/sickle). Considering that the span of paired-end reads might be larger than the length of library fragments, SeqPrep was applied for merging overlapped reads before further analysis (https://github.com/jstjohn/SeqPrep). Soybean genome and annotation files were downloaded from Phytozome (https://phytozome.jgi.doe.gov, Gmax V10) [48]. TopHat2 [49] with parameters “--library-type fr-firststrand -p 8” and Cufflinks [50] were utilized for genomic mapping, transcript recognition and calculation of normalized expression values.

For lncRNA identification, a stringent step-by-step filtration procedure was established for entire transcripts (Fig. 1). Based on the information of genomic locus, customized scripts were written for extracting non-overlapped transcripts against known genes in strand-specific mode. GtRNAdb [51], SILVA [52], Rfam [53] and miRBase [54] databases were used to eliminate house-keeping RNAs and soybean miRNA precursors. The remaining transcripts were subjected to CPC program [55] for predicting their coding potentials, and those with CPC score > 0.5 were filtered out. Then, the Swiss-Prot database [56] was applied for a double check and further removal of protein coding transcripts (Blastn, 1e-5). Low expressed (FPKM < 2 under both conditions) and short transcripts (< 200 nt) were also discarded. Finally, transcripts located on the opposite strands of known genes were classified as lncNATs, while novel intergenic transcripts 200 nt away from coding genes were deemed as lincRNAs. AgriGO [57], an integrated web-based GO analysis toolkit, was employed for functional annotation and enrichment analysis. For inter-species conservation analysis, wild soybean (*G. soja*, GCA_002907465.1) and chickpea (*C. arietinum*, GCF_000331145.1) genomes were downloaded from NCBI. Other plant genomes were downloaded from Phytozome.

### Identification of lncRNAs by RT-PCR

The first-strand complementary DNA (cDNA) synthesis was synthesized with random hexamer primer using the RevertAid First Strand cDNA Synthesis Kit (Thermo Scientific). The lncRNA cDNAs were amplified as templates with appropriate primers (Additional file 17: Table S11) by using the Platinum SuperFi Green PCR Master Mix (Thermo Scientific). Glyma11g33560 contained one intron which was set as control and amplified using genomic DNA and cDNA, respectively. RT-PCR products were detected by electrophoresis on 3% agarose gel. Twelve of these RT-PCR products were randomly selected and confirmed by Sanger sequencing.

### DNA methylation data analysis

Trimmomatic (v0.35) [58] was used to clean raw reads by removing adapter sequences and low-quality nucleotides with default parameters. Bismark (v0.16.3) [59] was employed for genomic alignment against converted reference genomes (C -> T or G -> A) and determining the methylation calls for each cytosine in CpG, CHG, and CHH contexts. For each gene or region, RMCs was calculated by dividing methylated cytosines by the total number of cytosines.

## Supplementary information

**Additional file 1: Figure S1.** Seed germination (A) and root tissues of soybean samples (C) cultured under control (B) and continuous salt stress (D) conditions.

**Additional file 2: Figure S2.** GO analysis of predicted target genes for lincRNAs (A) and lncNATs (B).

**Additional file 3: Figure S3.** Conservation analysis of lincRNAs and lncNATs among plant genomes.

**Additional file 4: Figure S4.** RMCs of transcripts (A) and base composition analysis (B) for CpG, CHG and CHH contexts.

**Additional file 5: Figure S5.** Experimental validation of 22 randomly chosen lincRNAs that came from repetitive regions by reverse transcription PCR.

**Additional file 6: Figure S6.** Expression levels (A) and CpG ratios (B) of lncRNAs and their targets under control and continuous salt stress.

**Additional file 7: Table S1.** Overview of whole transcriptome and methylation sequencing.

**Additional file 8: Table S2.** Transcriptome assemblies under different conditions.

**Additional file 9: Table S3.** Identified lincRNAs and the nearest genes from soybean roots.

**Additional file 10: Table S4.** Identified lncNATs and their antisense genes from soybean roots.

**Additional file 11: Table S5.** Sequence comparison between identified lncRNAs and soybean TEs.

**Additional file 12: Table S6.** Statistics of bisulfite sequencing and methylated cytosines.

**Additional file 13: Table S7.** Methylation ratios of lncRNAs and their promotor regions for CpG contexts.

**Additional file 14: Table S8.** Methylation ratios of lncRNAs and their promotor regions for CHG contexts.

**Additional file 15: Table S9.** Methylation ratios of lncRNAs and their promotor regions for CHH contexts.

**Additional file 16: Table S10.** FPKM values and methylation status for lncRNA target genes.

**Additional file 17: Table S11.** Primers for reverse transcription PCR experiment.

## Data Availability

Entire raw data generated from Illumina sequencing platform in this study, including whole transcriptome and bisulfite sequencing, were deposited in the Sequence Read Archive (SRA) database under accession No. PRJNA515384.

## Abbreviations

CDS: Protein coding regions
CMT: Chromomethylase
CPC: Coding Potential Calculator
CTAB: Cetyltrimethylammonium bromide
DRM2: Domain Rearranged Methyltransferase 2
FPKM: Fragments Per Kilobase per Million mapped reads
GO: Gene Ontology
incRNA: Intronic ncRNA
lincRNA: Long intergenic non-coding RNA
lncNAT: Natural antisense transcript
lncRNA: Long non-coding RNA
LTR: Long terminal repeat
MET1: Methyltransferase 1
MF: Molecular Function
miRNA: MicroRNA
NGS: Next-generation sequencing
RdDM: RNA-directed DNA methylation
RMCs: Ratios of methylated cytosines
RT-PCR: Reverse transcription PCR
SA: High salinity condition
TE: Transposable element
UTR: Untranslated region
WT: Water/control condition
