# Supplementary material for: Continuous salt stress-induced long non-coding RNAs and DNA methylation patterns in soybean roots
Source: BMC Genomics. 2019 Oct 12;20:730. doi: 10.1186/s12864-019-6101-7 (PMC6790039; doi:10.1186/s12864-019-6101-7)

Figure S1. Seed germination (A) and root tissues of soybean samples (C) cultured under control (B) and continuous salt stress (D) conditions.

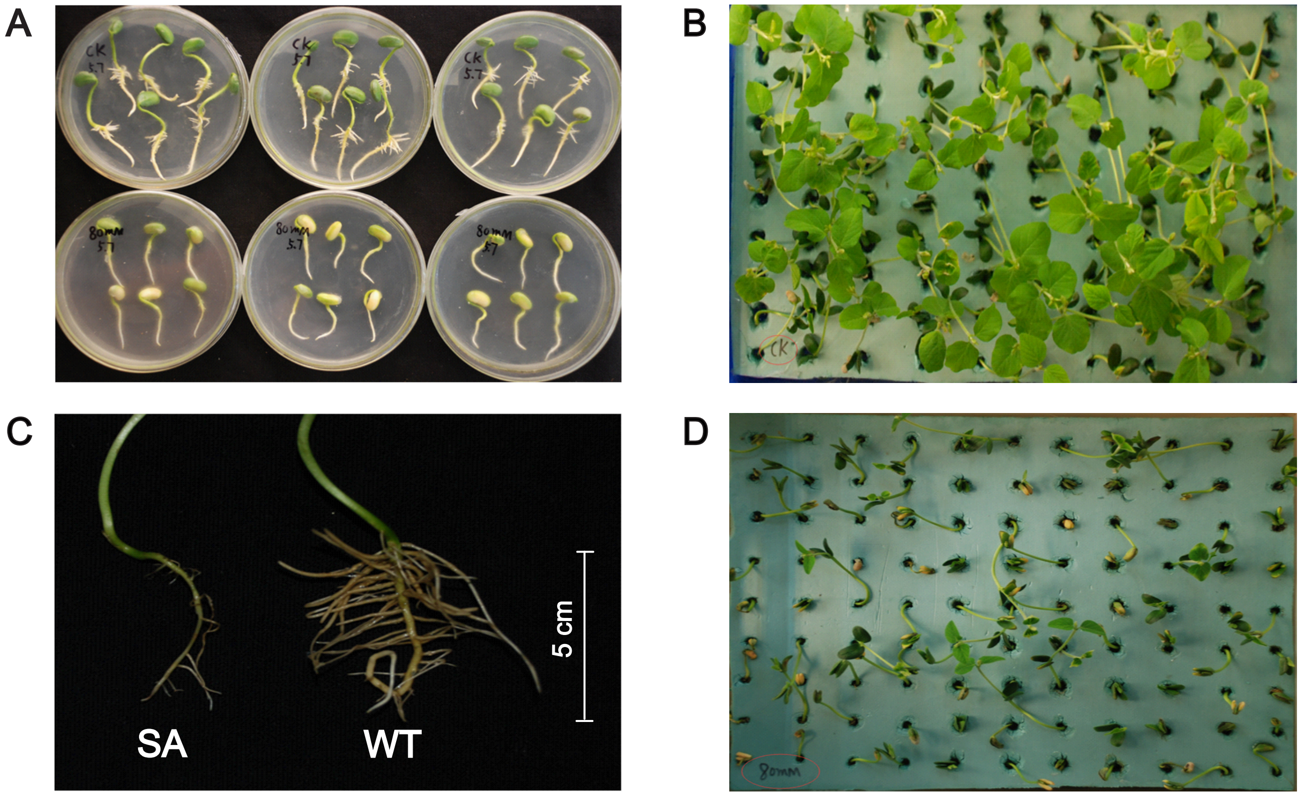

Supplement: Supplementary file 1 — Additional file 1: Figure S1. Seed germination (A) and root tissues of soybean samples (C) cultured under control (B) and continuous salt stress (D) conditions. [file 12864_2019_6101_MOESM1_ESM.pdf]
