# Supplementary figures and images for: Continuous salt stress-induced long non-coding RNAs and DNA methylation patterns in soybean roots
Source: BMC Genomics. 2019 Oct 12;20:730. doi: 10.1186/s12864-019-6101-7 (PMC6790039; doi:10.1186/s12864-019-6101-7)

Figure S2. GO analysis of predicted target genes for lincRNAs (A) and lincNATs (B).

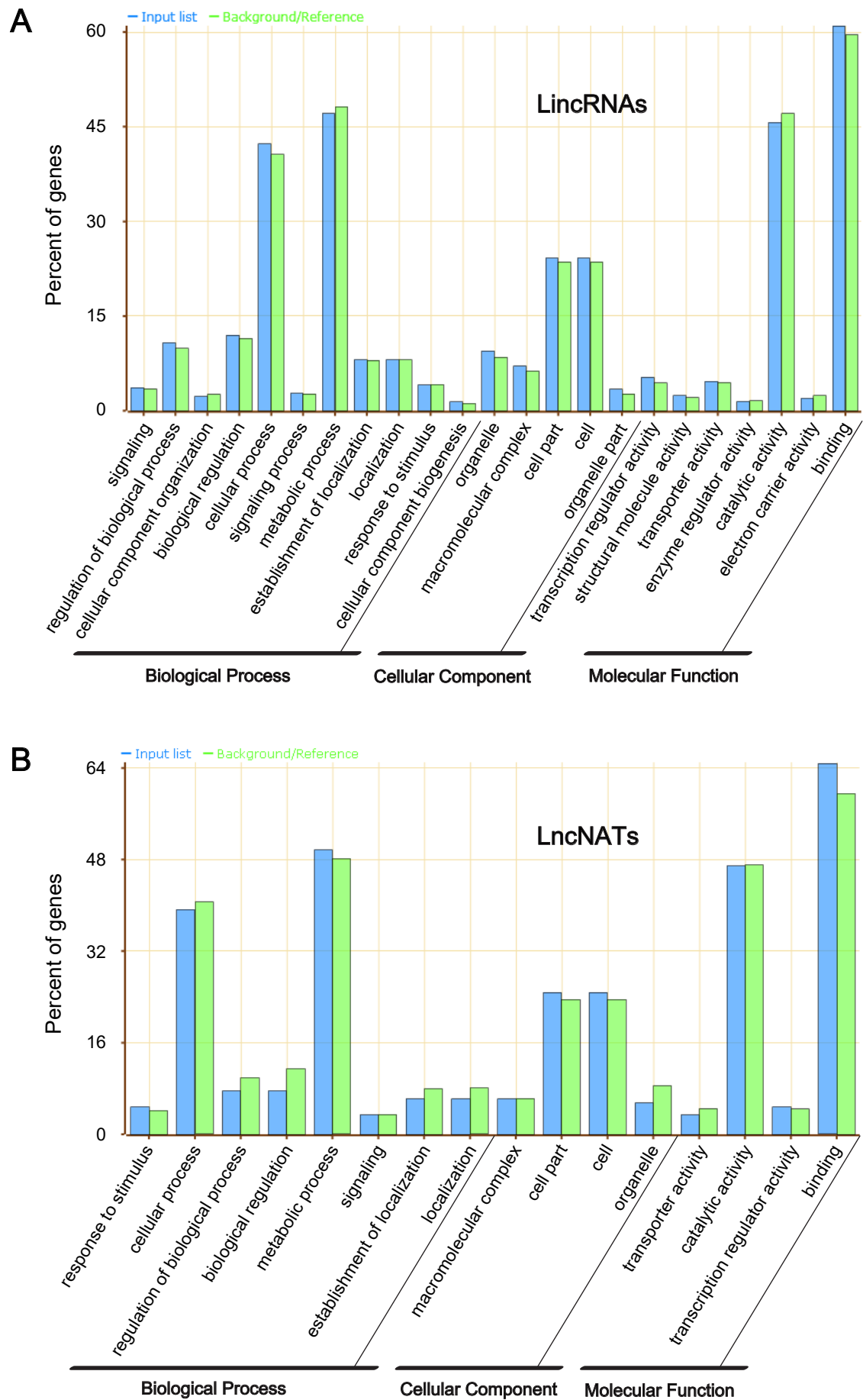

Supplement: Supplementary file 2 — Additional file 2: Figure S2. GO analysis of predicted target genes for lincRNAs (A) and lncNATs (B). [file 12864_2019_6101_MOESM2_ESM.pdf]

Figure S3. Conservation analysis of lincRNAs and lncNATs among plant genomes.

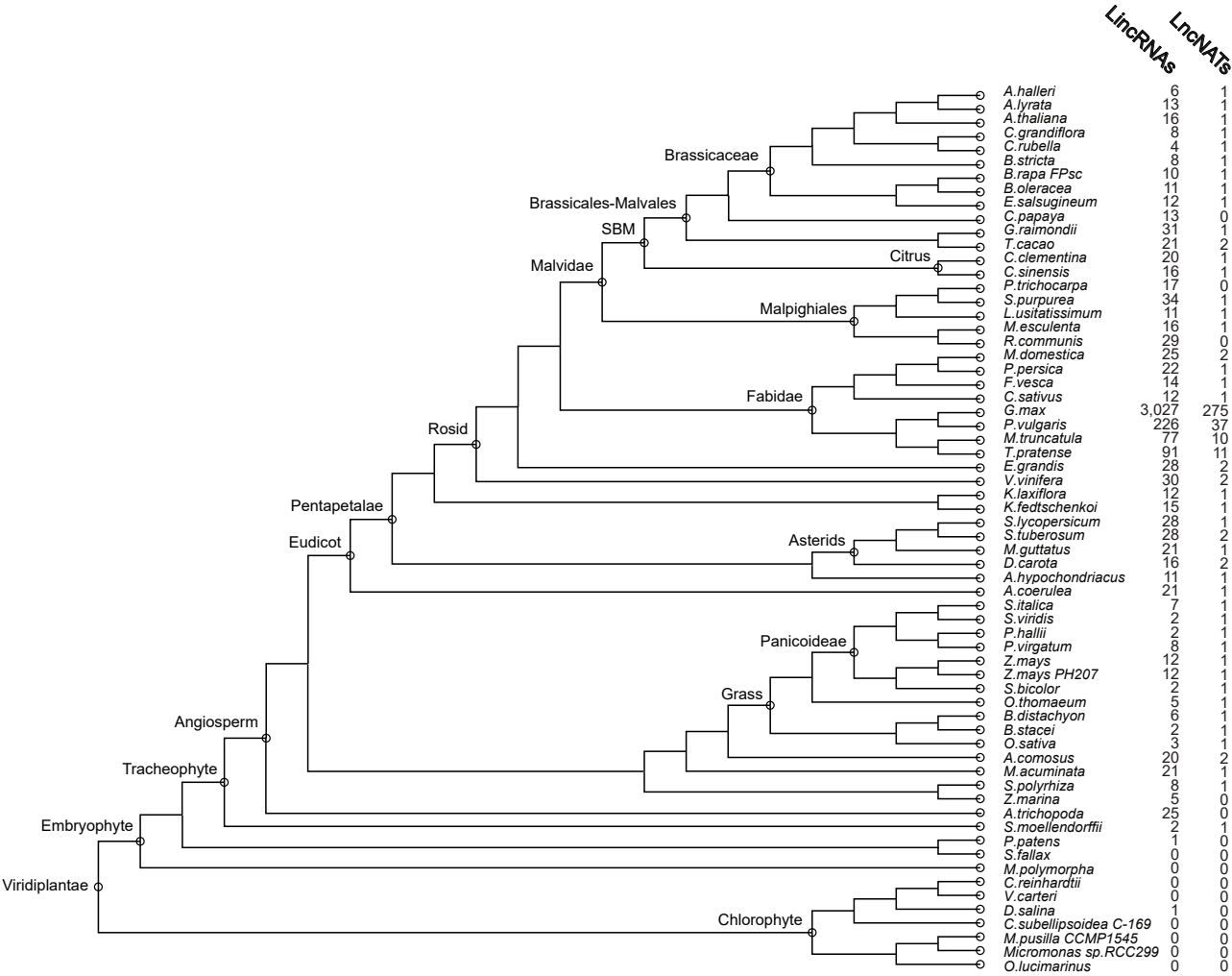

Supplement: Supplementary file 3 — Additional file 3: Figure S3. Conservation analysis of lincRNAs and lncNATs among plant genomes. [file 12864_2019_6101_MOESM3_ESM.pdf]

Figure S4. RMCs of transcripts (A) and base composition analysis (B) for CpG, CHG and CHH contexts.

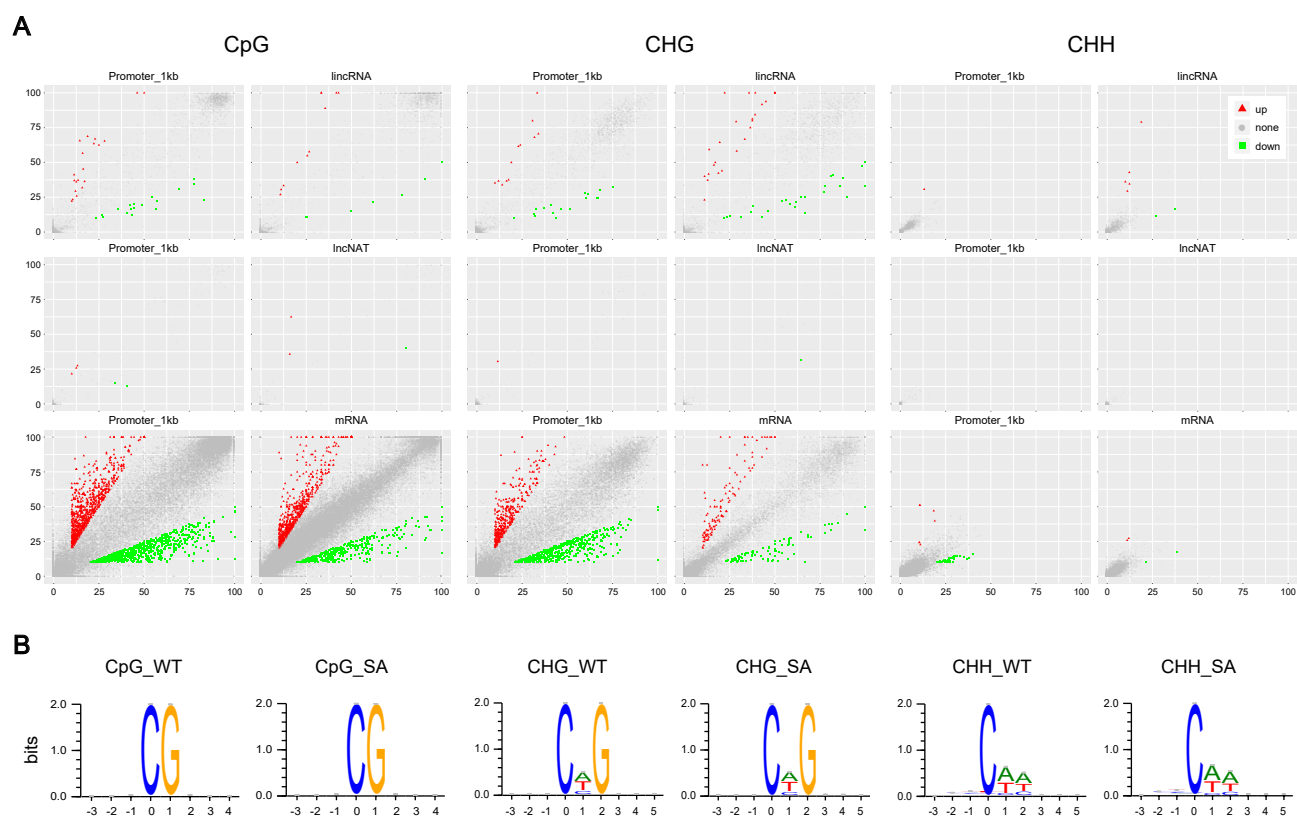

Supplement: Supplementary file 4 — Additional file 4: Figure S4. RMCs of transcripts (A) and base composition analysis (B) for CpG, CHG and CHH contexts. [file 12864_2019_6101_MOESM4_ESM.pdf]
