# Supplementary material for: Continuous salt stress-induced long non-coding RNAs and DNA methylation patterns in soybean roots
Source: BMC Genomics. 2019 Oct 12;20:730. doi: 10.1186/s12864-019-6101-7 (PMC6790039; doi:10.1186/s12864-019-6101-7)

Figure S5. Experimental validation of 22 randomly chosen lincRNAs that came from repetitive regions by reverse transcription PCR.

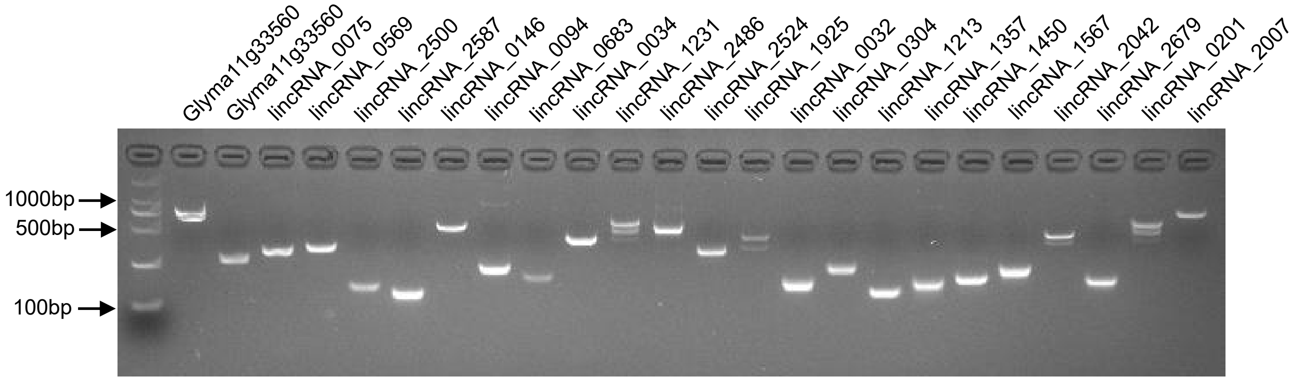

Supplement: Supplementary file 5 — Additional file 5: Figure S5. Experimental validation of 22 randomly chosen lincRNAs that came from repetitive regions by reverse transcription PCR. [file 12864_2019_6101_MOESM5_ESM.pdf]
