# Supplementary material for: Continuous salt stress-induced long non-coding RNAs and DNA methylation patterns in soybean roots
Source: BMC Genomics. 2019 Oct 12;20:730. doi: 10.1186/s12864-019-6101-7 (PMC6790039; doi:10.1186/s12864-019-6101-7)

Figure S6. Expression levels (A) and CpG ratios (B) of lncRNAs and their targets under water and continuous salt stress.

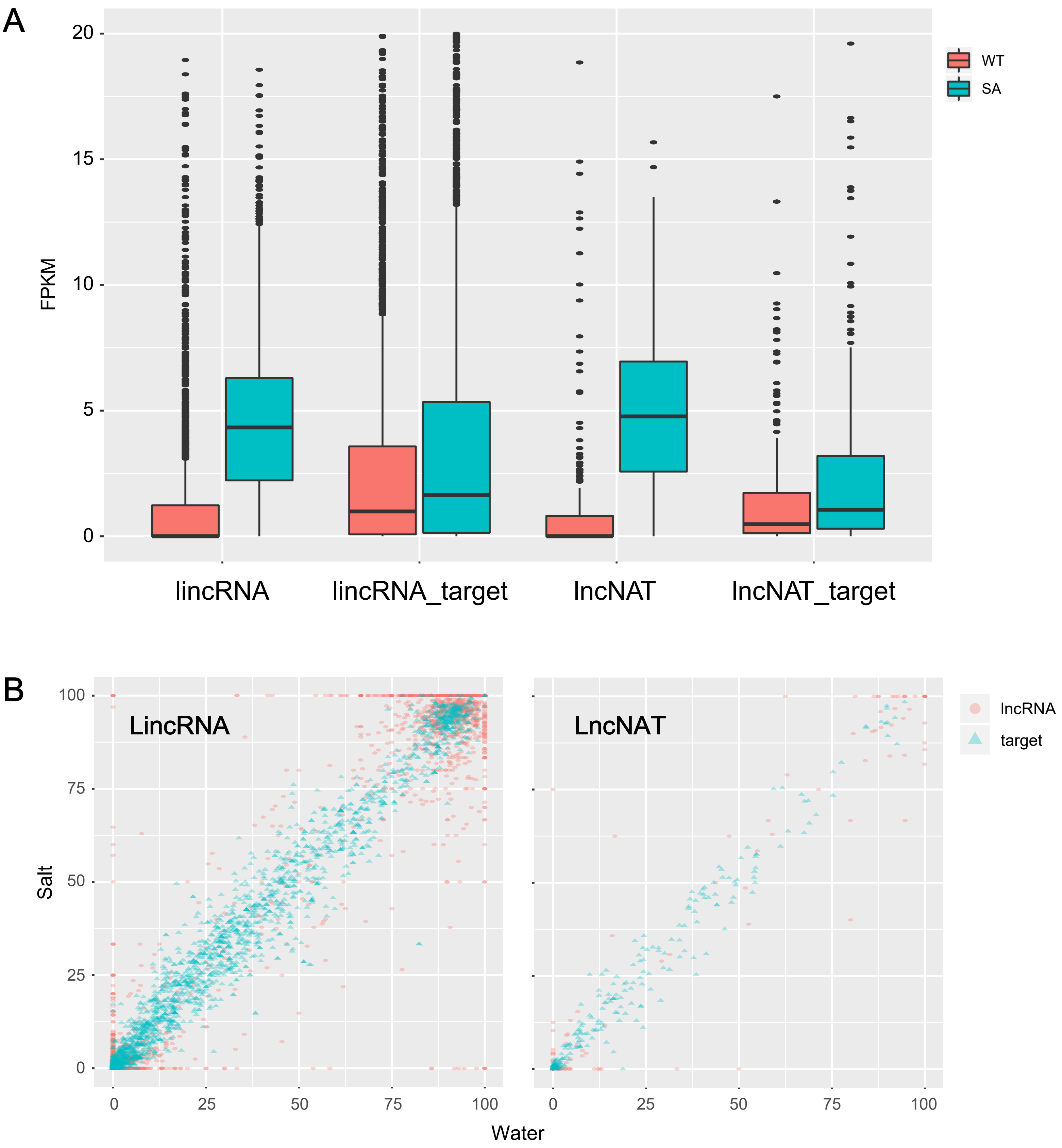

Supplement: Supplementary file 6 — Additional file 6: Figure S6. Expression levels (A) and CpG ratios (B) of lncRNAs and their targets under control and continuous salt stress. [file 12864_2019_6101_MOESM6_ESM.pdf]
